# Editorial: Fundamentals and applications of biomimetic materials

**DOI:** 10.3389/fbioe.2024.1447154

**Published:** 2024-06-26

**Authors:** Yi-Chen Ethan Li, Tooru Ooya, Jeng-Shiung Jan, Hung-Yin Lin

**Affiliations:** ^1^ Department of Chemical Engineering, Feng Chia University, Taichung, Taiwan; ^2^ Department of Chemical Science and Engineering, Graduate School of Engineering, Kobe University, Kobe, Japan; ^3^ Department of Chemical Engineering, National Cheng Kung University, Tainan, Taiwan; ^4^ Department of Chemical and Materials Engineering, National University of Kaohsiung, Kaohsiung, Taiwan

**Keywords:** fundamentals and applications, biomimetic materials, 3D printing, biomaterials, tissue engineering, virus-like particles

Biomimicry involves taking inspiration from nature by examining its models, systems, processes, and elements to solve human problems. Examples include the water repellency of shark skin (Fan et al., 2022), the strength of spider silk (Arndt et al., 2022), and the thermal insulation of honeycomb structure (Heng et al., 2013). This new science is based on the belief that nature follows the path of least resistance. This Research Topic collects high-quality research on biomimetic composite materials that are structurally or functionally similar to their biological equivalents for solving real-life scientific problems. In recent decades, the synthesis and applications of biomimetic materials, including composite materials, have become a rapidly growing and highly promising field.

Roldán et al. control the gelatin concentration, the diameter and surface roughness of gelatin nanofiber-based scaffold to mimic different tissue mechanical properties, such as gingiva, liver, and etc., through electrospinning. Gelatin was electrospun in nine different concentrations onto a rotating collector (Roldán et al.). They found that fiber diameter and interdistance were related to solvent concentration, which also significantly affected mechanical properties (Roldán et al.). These findings offer a solid foundation for future research to optimize factors using both traditional statistics and machine learning techniques for developing specific human tissue (Roldán et al.).

Wu et al. investigated the effects of polyvinyl alcohol (PVA) substrates blended with varying concentrations of collagen and/or gelatin on stem cell adhesion, proliferation, shape, spreading, and differentiation. They observed that fibroblasts could switch between oval, spindle, and flattened shapes depending on the collagen/gelatin concentration (Wu et al.). Additionally, neural stem cell differentiation was influenced by collagen concentration in the PVA substrates. These findings offer a versatile platform for controlling cell behavior *in vitro* using biomimetic scaffolds, with potential applications in regenerative medicine and tissue engineering (Wu et al.).

Inspired by the dihydroporphyrin structure coordinated with magnesium in chlorophyll and the iron-coordinated heme found in nature, Qiu et al. utilized the fact that porphyrins usually exist in the form of complexes with metal ions to develop a new type of end-functionalized porphyrin-derived photosensitizers. To address this, they prepared a novel photochemical and thermoresponsive diblock biomaterial with end-functionalized zinc porphyrin [(ZnPor-PAzo)–PNIPAM] and evaluated its photocatalytic activity for methylene blue (MB) in water (Qiu et al.). Their study demonstrated that the diblock copolymer (ZnPor-PAzo)–PNIPAM shows photocatalytic activity for aqueous MB under visible light (Qiu et al.).

He et al. designed and prepared porous PDMS films with regular surface patterns using 3D printing. Unlike conventional chemical foaming or physical pore formation, 3D printing ensures controllable and regular physical structures. They investigated the effects of printing speed and glass substrate surface wettability on PDMS filament morphology and further optimized the printing speed and number of layers for regulating both film morphology and surface wettability (He et al.). Their study demonstrated that 3D printing can easily fabricate regularly patterned porous PDMS films with specific, controllable surface wettability, offering a new method for creating such films to mimic the specific wettability of tissue surface (He et al.).

Finally, HIV Gag biomimetic virus-like particles (VLPs) are promising HIV vaccine candidates. However, low shear rates in tangential flow filtration limit VLP concentration efficiency. Wolf et al. investigated the effect of high shear rates on the colloidal stability of mosaic VLPs (Mos-VLPs), relevant to HIV Gag VLPs. They found that Mos1.Gag + Mos2S.Env VLPs (eVLPs—with envelope proteins) exhibited higher colloidal stability, with increased the average hydrodynamic diameter and the polydispersity index during storage at high shear rates, compared to HIV Mos1.Gag VLPs (bVLPs—without envelope proteins) (Wolf et al.). Additionally, they confirmed that the dispersion medium significantly impacts the stability of Mos-VLPs (Wolf et al.).

In conclusion, we thank the participants for their contributions. We hope this Research Topic aids the development of novel biomimetic materials for biomedical applications.

## References

[B1] Arndt T. Greco G. Schmuck B. Bunz J. Shilkova O. Francis J. (2022). Engineered spider silk proteins for biomimetic spinning of fibers with toughness equal to dragline silks. Adv. Funct. Mater 32 (23), 2200986. 10.1002/adfm.202200986 36505976 PMC9720699

[B2] Fan S. Han* X. Tang Y. Wang Y. Kong X. (2022). Shark skin—an inspiration for the development of a novel and simple biomimetic turbulent drag reduction topology. Sustainability 14 (24), 16662. 10.3390/su142416662

[B3] Heng L. Wang B. Li M. Zhang Y. Jiang L. (2013). Advances in fabrication materials of honeycomb structure films by the breath-figure method. Mater. (Basel) 6 (2), 460–482. 10.3390/ma6020460 PMC5452082 28809319

